# Supplementary material for: A “Mammalian-like” Pycnodont Fish: Independent Acquisition of Thecodont Implantation, True Vertical Replacement, and Carnassial Dentitions in Carnivorous Mammals and a Peculiar Group of Pycnodont Fish
Source: Life (Basel). 2022 Feb 8;12(2):250. doi: 10.3390/life12020250 (PMC8878644; doi:10.3390/life12020250)

life

Supplementary Material S1

Systematic Paleontology

Osteichthyes Huxley, 1880

Actinopterygii Cope, 1887

Pycnodontiformes Berg, 1937

Serrasalmimidae Vullo et al., 2017

Diagnosis presence of monocuspid or bicuspid mammiform teeth with cingulum, most or all teeth of main rows elevated, longer and higher than wide

Serrasalmimus secans Vullo et al., 2017

Locality–Ouled Abdoun couche II, Morocco

Specimen– NMNS-PV20561, a right vomer with six teeth (Figure 1, S1–2). This fossil was purchased by the National Museum of Nature and Science, Japan, from the Stone Age (Tokyo International Mineral Association) in June 2002. It was originally misidentified as "a right upper jaw of Mammalia indet." upon purchase. We corrected it to a fragmentary right vomer of the pycnodont fish S. secans owing to a detailed study by Vullo et al. [5].

Diagnosis–A shear-type dentition, small mammiform teeth, strongly flattened, labiolingually compressed prearticular bone, prearticular dentition consisting of one row.

Description– NMNS PV-20561 is a fragmentary right vomer with six teeth located in the anterior portion of the bone. The measurement references are mentioned in Figure S3, and the data are listed in Table S1–2. All teeth are compressed labio-lingually and aligned on a straight row with minor overlapping between the widest part of the sequential teeth. The labial sides of the teeth are flat, showing a large area of smooth wear surface caused by sliding contact with the lower dentition. All the preserved teeth are clearly bicuspid and aligned anteroposteriorly with the anterior cusp smaller than the posterior one. In three of the teeth positioned posteriorly, the anterior cusp is 73% of the size of the posterior cusp. The anterior cusps are smaller in the other teeth positioned anteriorly, but their relative sizes to the posterior cusps are unknown due to the lack of either cusp. The posterior cusp is slightly inclined to the vertical axis of the root-like structure, whereas the anterior cusp is along the axis of the root-like structure or only slightly inclined to it. The labial side of the enameloid is abraded heavily towards the apices of the cusps. The enameloid is thickened on the base of the crown to form a cingulum on the lingual side. The cingulum is more developed in the teeth positioned posteriorly in comparison to the anteriorly developed ones. There is no cingulum developed on the labial side.

Discussion about eruption of the replacement teeth in Serrasalmimus secans

An important question would be whether or not the timing of tooth replacement differs among different loci of the functional teeth. If all the functional teeth are lost as a unit and replaced simultaneously, as in the case of extant serrasalmid fish (e.g., piranhas, Kolmann et al., 2019), a correlation between in size of the functional and replacement teeth may be hypothesized. Therefore, we examined the size distributions of the functional teeth and replacement teeth and correlations between them. Measurements points were shown in Figure S3, and measurements results are listed in Table S2.

The correlations between the size of functional and replacement teeth are shown in Figure S4. The volume of the functional teeth has no size trend, but the volume of replacement teeth is divided into two size groups. The crown length divides the dentition of functional teeth into two size groups and that of replacement teeth into three groups. The crown height shows the same pattern as its width in the functional teeth, whereas they are different in the replacement teeth. The crown height of the functional teeth was similar to its tubular root height. These linear measurements of the functional teeth are significantly correlated with the volume of the functional teeth and that of the replacement teeth. However, there is no correlation between the tooth volume and linear measurements in the replacement teeth. In addition, no correlation was found between the linear measurements of the functional teeth

and replacement teeth except for the root of the functional teeth vs. the width of the replacement teeth and the crown height of the functional teeth vs. the height of the replacement teeth. This suggests that the teeth measurements are good proxies for the volume of the functional teeth. On the other hand, the measurements of the replacement teeth do not reflect their volume. Importantly, no size correlation was noticed between the functional and replacement teeth of the same number, suggesting that the tooth row of S. secans would not be shed simultaneously but replaced at different timings.

Abbreviation

NMNS, National Museum of Natural History, Ibaraki, Japan.

Supplementary references

Huxley, T. H. 1880. On the Application of the Laws of Evolution to the Arrangement of the Vertebrata and, more particularly, of the Mammalia. pp. 649.

Cope, E.D. 1887. Geology and paleontology. The American Naturalist, 21, 1014-1019

Berg, L.S. 1937. A classification of fish-like vertebrates. Bulletin de l'Académie Des Sciences de l'URSS, 4, 1277–1280.

Vullo, R., Cavin, L., Khalloufi, B., Amaghzaz, M., Bardet, N., Jalil, N. E., Jourani E., Khaldoune, F. and Gheerbrant E. 2017. A unique Cretaceous-Paleogene lineage of piranha-jawed pycnodont fishes. Scientific reports, 7, 6802.

Table S1. Measurement of the whole size of NMNS PV-20561. (fossil measurements are shown in Figure S3 A).

measurements	
Length	*35.63 mm*
Width	*5.92 mm*
Height	*15.07 mm*

Table S2. Tooth measurements of teeth of NMNS PV-20561.

	Functional Teeth						***Replacement Teeth***			
Tooth number	*Volume*	*Crown length*	*Crown width*	*Crown height*	*Root length*	*Total height*	*Volume*	*Width*	*Length*	*Height*
1	*38.69*	*4.78*	*2.94*	*2.15*	*4.11*	*6.26*	*1.67*	*2.83*	*1.17*	*1.82*
2	*56.12*	*4.56*	*3.36*	*3.74*	*4.16*	*7.90*	*1.13*	*2.73*	*0.95*	*1.52*
3	*69.89*	*5.52*	*3.36*	*3.76*	*4.62*	*8.38*	*3.69*	*3.27*	*1.00*	*3.01*
4	*84.71*	*5.58*	*3.72*	*5.52*	*4.56*	*10.08*	*6.15*	*3.43*	*1.16*	*3.75*
5	*91.84*	*5.48*	*3.94*	*5.74*	*4.45*	*10.19*	*4.49*	*2.81*	*1.30*	*2.85*
6	*92.20*	*5.66*	*3.84*	*4.96*	*4.73*	*9.69*	*5.31*	*3.31*	*1.67*	*2.66*

Figure S1 A 3D model of the right vomer of Serrasalmimus secans from the Phosphorite Bed II (Thanetian age, Paleocene) in the Ouled Abdoun Basin, Morocco. A: Ventral view, B: medial view, C: lateral view, D: dorsal view, E: posterior view, F: anterior view.

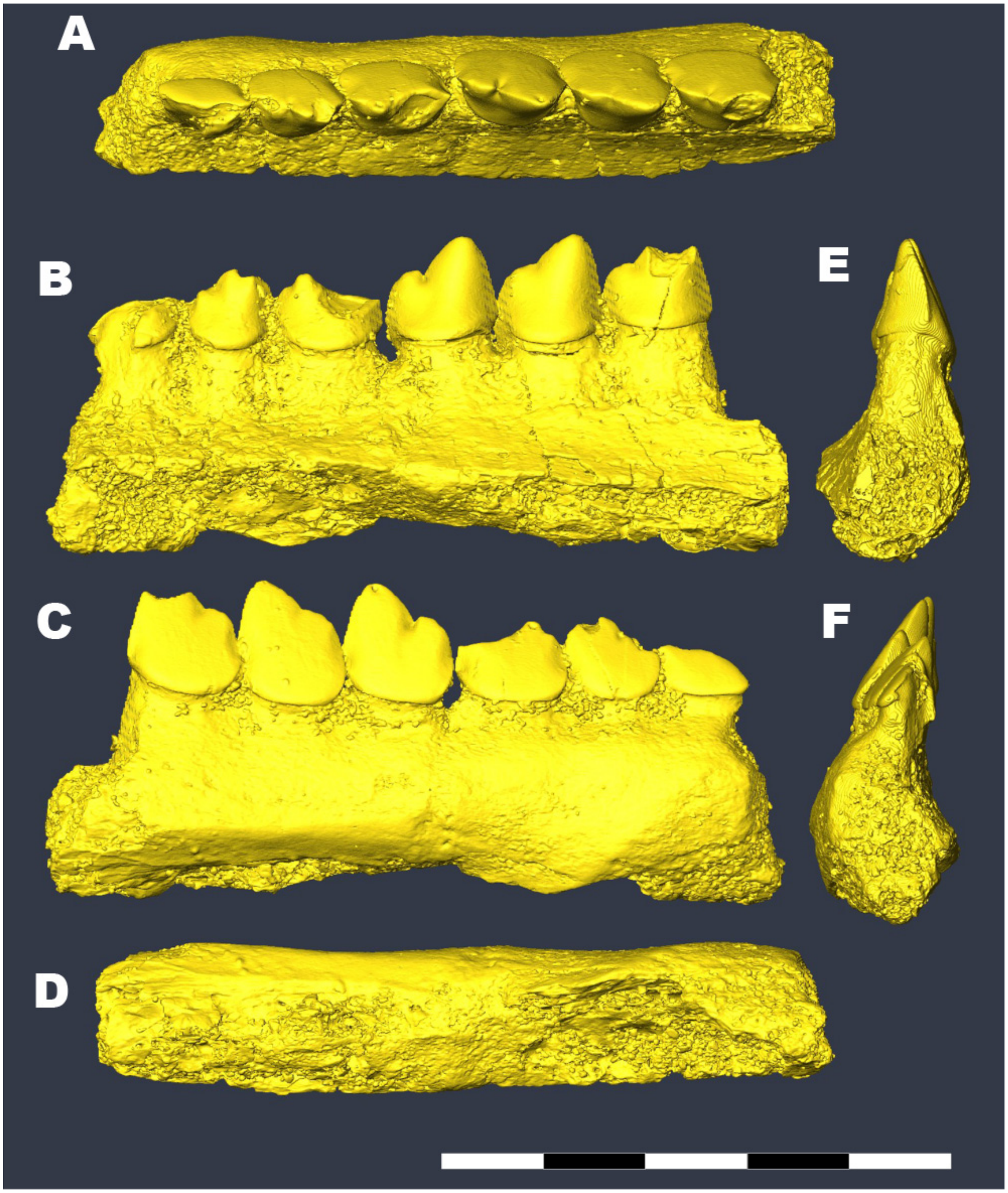

Figure S2 A 3D model of all functional teeth. The numbers are consistent with those mentioned in Table S2 and Figure S3.

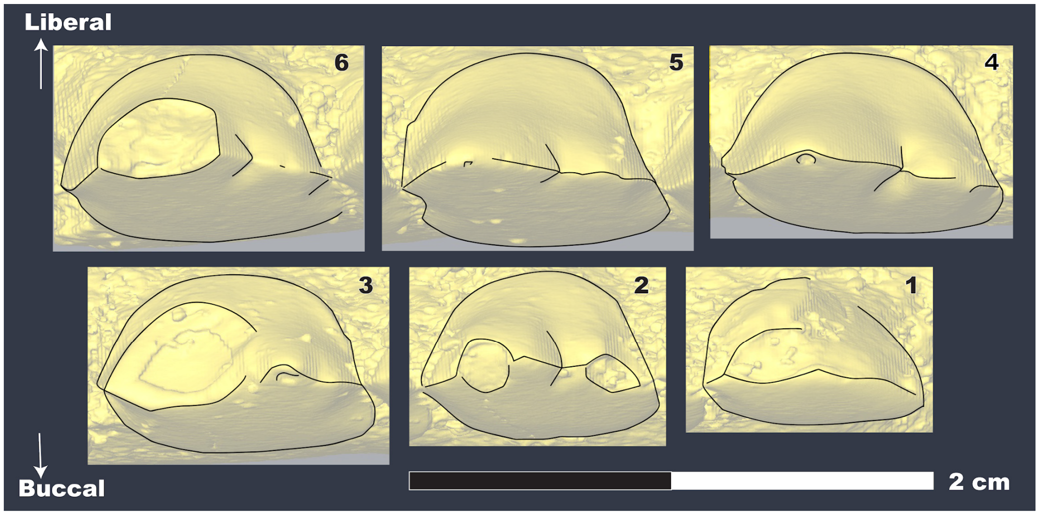

Figure S3 A diagram of NMNS PV-20561 showing the measurements of (A) whole fossil and (B) teeth.

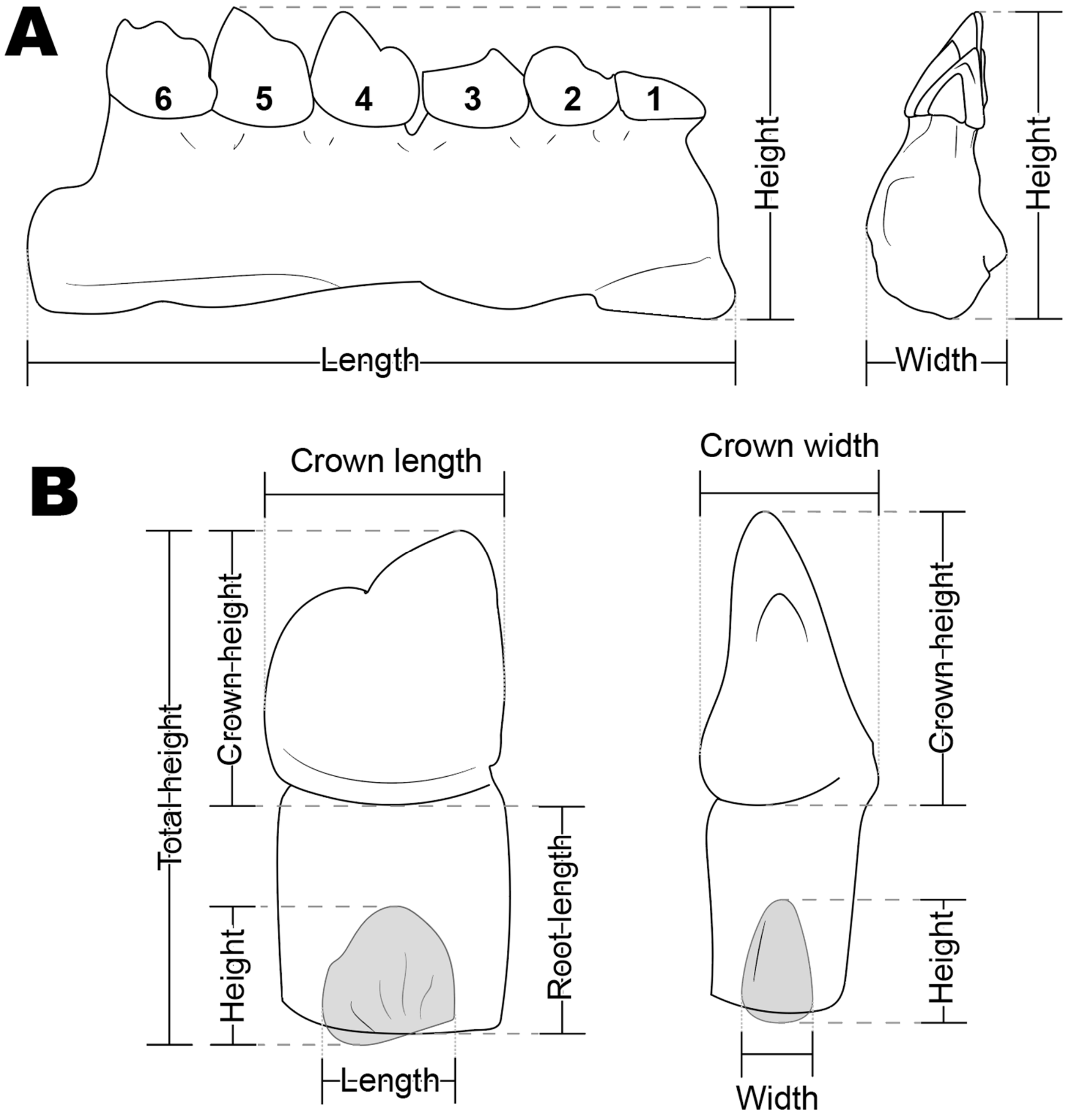

Figure S4. Size distribution and correlation coefficients of the functional and replacement teeth of NMNS PV-20561.

Highlighted in yellow: strongly correlated and p<0.05

Volume: functional teeth volume, Crown.length: measurements of the crown length of functional teeth, Crown.width: measurements of crown width of functional teeth, Crown.height: measurements of the crown height of functional teeth, Root.length: measurements of root length of functional teeth, Total.height: measurements of maximum length of functional teeth, Volume.1: volume of replacement teeth, Width: measurements of the maximum width of replacement teeth, Length: measurements of maximum length of replacement teeth, Hight: measurements of maximum height of replacement teeth.

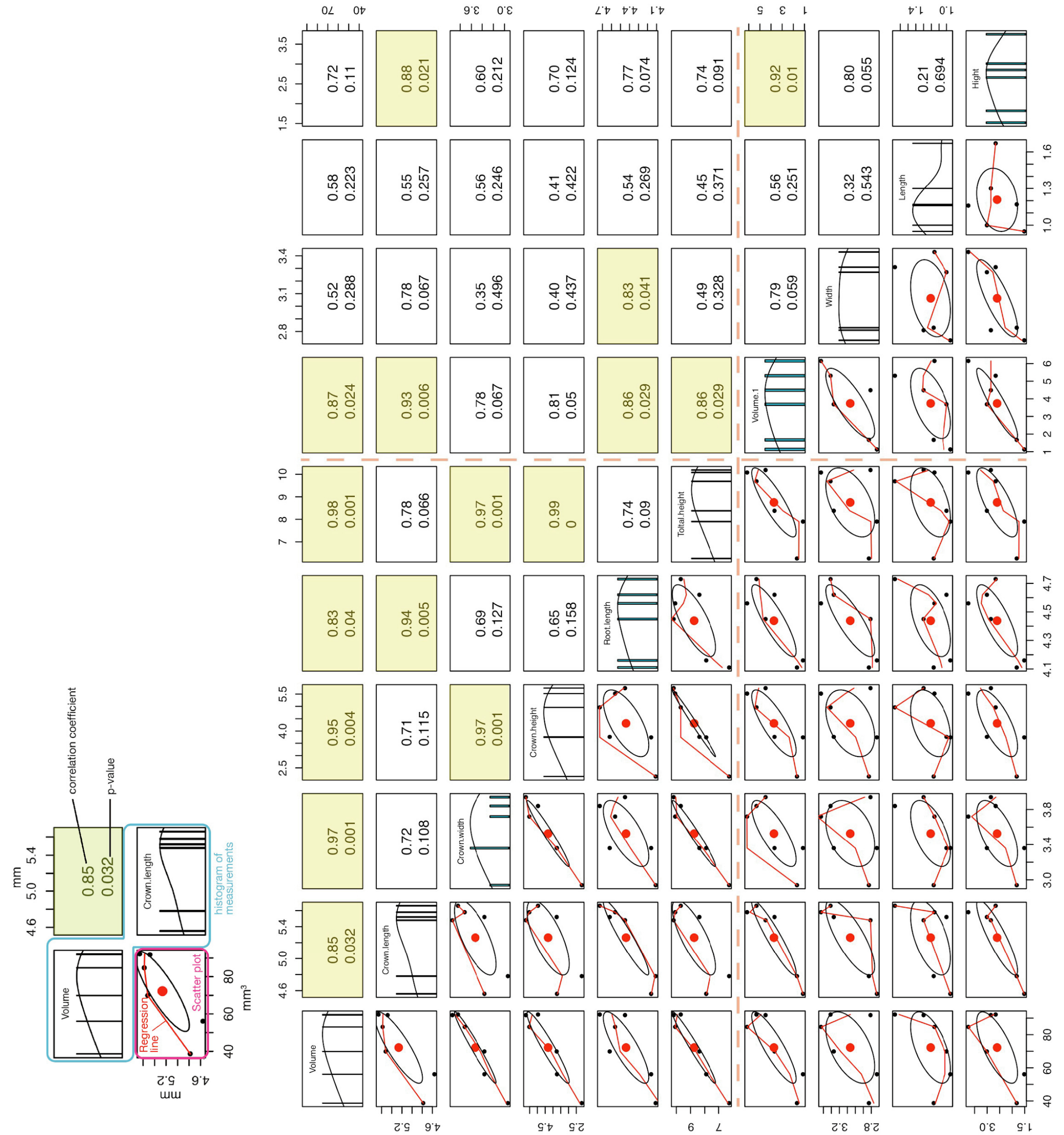

Supplement: Supplementary file 1 [file life-12-00250-s001.zip › Supplementary Material S1-life-1591663.pdf]
